# Increased default mode network activation in depression and social anxiety during upward social comparison

**DOI:** 10.1093/scan/nsaf012

**Published:** 2025-01-30

**Authors:** Alejo Acuña, Sebastián Morales, Laura Uriarte-Gaspari, Nara Aguirre, Antonella Brandani, Natalia Huart, Javier Mattos, Alfonso Pérez, Enrique Cuña, Gordon Waiter, Douglas Steele, Jorge L Armony, Margarita García-Fontes, Álvaro Cabana, Victoria B Gradin

**Affiliations:** Center for Basic Research in Psychology (CIBPsi), Facultad de Psicología, Universidad de la República, Montevideo 11200, Uruguay; Sección Neurociencias, Facultad de Ciencias, Universidad de la República, Montevideo 11400, Uruguay; Center for Basic Research in Psychology (CIBPsi), Facultad de Psicología, Universidad de la República, Montevideo 11200, Uruguay; Center for Basic Research in Psychology (CIBPsi), Facultad de Psicología, Universidad de la República, Montevideo 11200, Uruguay; Center for Basic Research in Psychology (CIBPsi), Facultad de Psicología, Universidad de la República, Montevideo 11200, Uruguay; Center for Basic Research in Psychology (CIBPsi), Facultad de Psicología, Universidad de la República, Montevideo 11200, Uruguay; Hospital de Clínicas, Facultad de Medicina, Universidad de la República, Montevideo 11800, Uruguay; Centro Uruguayo de Imagenología Molecular (CUDIM), Montevideo 11600, Uruguay; Center for Basic Research in Psychology (CIBPsi), Facultad de Psicología, Universidad de la República, Montevideo 11200, Uruguay; Center for Basic Research in Psychology (CIBPsi), Facultad de Psicología, Universidad de la República, Montevideo 11200, Uruguay; Centro Uruguayo de Imagenología Molecular (CUDIM), Montevideo 11600, Uruguay; Aberdeen Biomedical Imaging Centre, University of Aberdeen, Aberdeen AB25 2ZN, United Kingdom; School of Medicine, University of Dundee, Dundee DD1 9SY, United Kingdom; Department of Psychology, McGill University, Montreal QC H3A 1G1, Canada; Centro Uruguayo de Imagenología Molecular (CUDIM), Montevideo 11600, Uruguay; Center for Basic Research in Psychology (CIBPsi), Facultad de Psicología, Universidad de la República, Montevideo 11200, Uruguay; Instituto de Fundamentos y Métodos en Psicología, Facultad de Psicología, Universidad de la República, Montevideo, Uruguay; CICADA, Centro Interdisciplinario de Ciencia de Datos y Aprendizaje Automático, Universidad de la República, Montevideo, Uruguay; Center for Basic Research in Psychology (CIBPsi), Facultad de Psicología, Universidad de la República, Montevideo 11200, Uruguay; Instituto de Fundamentos y Métodos en Psicología, Facultad de Psicología, Universidad de la República, Montevideo, Uruguay

**Keywords:** social comparison, depression, social anxiety, fMRI

## Abstract

Social comparisons are a core feature of human life. Theories posit that social comparisons play a critical role in depression and social anxiety triggering negative evaluations about the self, as well as negative emotions. We investigated the neural basis of social comparisons in participants with major depression and/or social anxiety (MD-SA, *n* = 56) and healthy controls (*n* = 47) using functional magnetic resonance imaging. While being scanned participants performed a social comparison task, during which they received feedback about their performance and the performance of a coplayer. Upward social comparisons (being worse than the coplayer) elicited high levels of negative emotions (shame, guilt, and nervousness) across participants, with this effect being enhanced in the MD-SA group. Notably, during upward comparison the MD-SA group showed greater activation than the control group in regions of the default mode network (DMN). Specifically, for upward comparison MD-SA participants demonstrated increased activation in the dorsomedial prefrontal cortex and reduced deactivation in the posteromedial cortex, regions linked to self-referential processing, inferences about other people’s thoughts, and rumination. Findings suggest that people with depression and social anxiety react to upward comparisons with a more negative emotional response, which may be linked to introspective processes related to the DMN.

## Introduction

Social comparison is a fundamental aspect of human life (Festinger 1954, Buunk and Gibbons 2007). When people make judgments about whether they are tall/short, rich/poor, or successful/losers, they do so in relative rather than absolute terms, comparing themselves to other people. Social comparisons are so inherent to social interactions that in order to avoid comparisons, one would have to isolate oneself from society (Swallow and Kuiper 1988). In the field of social comparisons, upward/downward comparisons refer to comparing oneself with someone considered to be better/worse than us. Upward comparisons can lead to learning and improving skills, but they can also be threatening to self-esteem, highlighting personal inadequacies and flaws, and eliciting negative emotions such as shame and envy (Luo et al. 2018). Conversely, downward comparisons can protect self-esteem and evoke positive emotions, but they can also lead to negative emotions such as guilt depending on the context (Buunk and Gibbons 2007, Luo et al. 2018).

Importantly, social comparisons are thought to play a key role in mental health disorders such as depression (Swallow and Kuiper 1988) and social anxiety (Antony et al. 2005). These disorders are characterized by negative self-evaluations and low self-esteem, with individuals being very critical of themselves and feeling worthless and inadequate (Beck et al. 1979, Clark and Wells 1995, Disner et al. 2011). It has been proposed that social comparisons can act as a precursor and reinforcer of these negative self-evaluations, contributing to the onset and maintenance of these disorders (Swallow and Kuiper 1988, Antony et al. 2005, McCarthy and Morina 2020).

Several studies have provided evidence that social comparisons can be especially challenging for individuals with depression and social anxiety. It has been shown that depressed individuals react to social comparisons by interpreting and recalling information in a less self-serving way (Buunk and Brenninkmeyer 2000, McCarthy and Morina 2020), focus on the fact that others are better than they are, reduce positive affect in a pronounced way when facing upward comparison (Bazner et al. 2006), and avoid social comparisons even if this leads to personal costs (Fernández-Theoduloz et al. 2019, Uriarte-Gaspari et al. 2022). Similarly, studies in social anxiety have shown that individuals with this disorder react to upward social comparisons with greater increases in negative affect compared to controls and that they are more likely to avoid interactions in order to avoid comparisons (McCarthy and Morina 2020, Uriarte-Gaspari et al. 2022).

Some studies have investigated the neural basis of social comparisons using functional magnetic resonance imaging (fMRI). It has been observed that downward comparisons activate regions implicated in reward processing such as the ventral striatum (Fliessbach et al. 2007, Dvash et al. 2010, Bault et al. 2011) and the medial prefrontal cortex (mPFC) (Bault et al. 2011, Lindner et al. 2015). On the other hand, upward comparisons activate regions linked to the experience of social pain, cognitive conflict, and salience detection such as the dorsal anterior cingulate cortex (ACC), the dorsomedial PFC, and the anterior insula (Takahashi et al. 2009, Kedia et al. 2014, Lindner et al. 2015, Luo et al. 2018).

Social comparisons have also been related to mentalizing and self-referential processes (Swencionis and Fiske 2014). This is because it is likely that social comparisons trigger inferences about other people’s thoughts (e.g. “she may be thinking she is better than me”) as well as thoughts about the self (e.g. “I’m not as good as others”). The mentalizing network (Schurz et al. 2014) and brain regions involved in self-referential processing (Northoff et al. 2006, Lemogne et al. 2012) overlap with the default mode network (DMN) (Padmanabhan et al. 2017, Davey and Harrison 2022). The DMN is thought to be critical for introspective thinking and it includes regions such as the mPFC and the posteromedial cortex [precuneus, posterior cingulate cortex (PCC), and retrosplenial cortex] (Raichle 2015). Numerous studies have shown that the DMN decreases its activation during tasks that imply attention to external stimuli. In contrast, the DMN activates in association to internal processes such as self-referential thinking, theory of mind, episodic memory, and mind wandering (Menon 2023). Activations in DMN regions, such as the mPFC observed in tasks that imply social comparisons, have been related to self-referential processing and mentalizing (Swencionis and Fiske 2014).

Interestingly, the DMN is thought to be relevant in internalizing disorders such as depression and social anxiety, which are characterized by excessive self-focus and rumination (Penney and Abbott 2014, Norton and Abbott 2016, LeMoult and Gotlib 2019). It has been proposed that these symptoms relate to abnormal dynamics between neural networks involved in the allocation of resources to the external and internal world (Fossati 2019, Yoon et al. 2019). In particular, an altered interaction between the DMN and attentional networks would lead to a bias to prioritize attention toward the internal world in these disorders, favoring self-focusing and rumination, at the expense of attending external stimuli. Consistent with this, several studies have reported increased activation of DMN regions in depression and social anxiety. In the case of depression, findings include increased ACC and precuneus activations in response to sad words (Mitterschiffthaler et al. 2008); increased PCC activation during negative distractors (Kaiser et al. 2015); a failure to reduce activity in the ventromedial PFC (vmPFC) and ACC while viewing passively and reappraising negative pictures (Sheline et al. 2009); increased mPFC activation for self-focusing (Lemogne et al. 2012); and decreased deactivation in the PCC and vmPFC in response to emotional stimuli [Grimm et al. 2009; see Borserio et al. (2021) for a review]. In the case of social anxiety, increased activation in the mPFC in response to self-referential criticism (Blair et al. 2008); increased responses to unintentional transgression in the vmPFC (Blair et al. 2010); increased activation in the mPFC during self-focused attention (Blair et al. 2011); increased responses to eye contact in the PCC (Schneier et al. 2011); reduced deactivation in the precuneus and PCC during emotion processing (Gentili et al. 2009); increased activation in the mPFC, precuneus, and PCC in response to disorder related visual scenes (Heitmann et al. 2016, 2017); and increased responses in the mPFC and PCC during memory encoding (Yoon et al. 2016) have been reported [see Yoon et al. (2019) for a review].

Despite the importance of social comparisons in depression and social anxiety, in terms of fMRI studies, to our knowledge there are no studies in depression and there is only one pilot study (Lin et al. 2023) in social anxiety. This previous study compared emotional and neural responses in participants with social anxiety vs. healthy controls, while they performed a choice task where they received performance feedback (correct, incorrect, and noninformative). Participants also received social comparison information related to whether a few, half, or most other players had also chosen the same option as the participant. Participants with social anxiety showed reduced activation in the bilateral putamen when the participant’s performance was inconsistent with the performance of most other players. As the putamen is part of the striatum that is associated with reward processing, the finding was interpreted as suggesting that social anxiety relates to reduced social rewards when performance feedback is inconsistent with the feedback of most other people. However, the social anxiety and the control groups did not differentiate in emotional ratings related to social comparison, possibly due to the small sample size, according to the authors.

Given the importance of social comparisons in disorders such as depression and social anxiety, plus the almost nonexistent research looking at the neural substrates of social comparisons in these disorders, our study aimed to contribute in this direction. We implemented a social comparison task where participants compared their performance in answering general knowledge questions with that of a coplayer. This task was used with fMRI to investigate emotional reports and brain responses to social comparison in healthy controls and participants with symptoms of depression and social anxiety. We expected that across participants, upward comparison (being worse than the coplayer) would be associated with negative emotions such as embarrassment and nervousness and that these negative emotions would be enhanced in participants with symptoms of depression and social anxiety.

In terms of neural activations, it was expected that depression and social anxiety could lead to altered reward brain responses during social comparisons. This is because, in the case of depression, a significant number of studies have shown abnormalities in brain reward signals, related to anhedonia symptoms (Keren et al. 2018, Borsini et al. 2020). Among the most consistent reward findings in depression is striatal hypoactivation. Social anxiety has also been related to low positive affect and abnormalities in reward processing (Carlton et al. 2020). While some studies have reported increased subcortical (including striatum) activity in social anxiety (Lorberbaum et al. 2004), several studies have reported reduced striatal activation in social anxiety, in particular while anticipating social rewards (Richey et al. 2014, 2017, Cremers et al. 2015), while performing being observed (Becker et al. 2017), during speech anticipation (Boehme et al. 2014), and, as mentioned earlier, in relation to social comparison (Lin et al. 2023). Therefore, based on this body of evidence, it was expected that participants with depression and social anxiety could show reduced reward responses in regions such as the striatum during social comparisons. In addition, since social comparisons are likely to engage self-focusing and rumination, with these processes being enhanced in depression and social anxiety, we hypothesized that participants with these disorders would show increased activation in DMN regions in relation to social comparison.

Of note, in our analysis we combined participants with depression, social anxiety, or both disorders in a single group. This was based on the fact that these disorders are highly comorbid (Koyuncu et al. 2019); share a similar affective profile of low positive and high negative affect, and in particular share cognitive features (Arditte Hall et al. 2019, Alvi 2020). Both disorders are associated with negative self-statements and negative interpretative and attentional biases. It has been proposed that there is a shared vulnerability underlying these disorders related to an excessive belief that one is not good enough (Langer and Rodebaugh 2014). This belief would be behind the feeling that one is of lower status compared to others. In relation to this, as presented earlier, similar hypotheses can be raised for both disorders regarding social comparisons. Based on this rationale, we compared healthy controls vs. a group of participants that qualify for depression, social anxiety, or both disorders. In addition, since in recent years it has been emphasized the importance of studying the neurobiology of mental health disorders in a continuous manner, along dimensions that span diagnostic boundaries (Cuthbert 2015, MacNamara et al. 2017), apart from comparing brain activity between groups, we implemented a dimensional analysis. We applied a principal component analysis (PCA) to the scores of the psychological questionnaires used in the study in order to describe the psychological characteristics of the sample in a more compact low-dimensional manner and to study brain activity from a dimensional perspective.

## Materials and methods

### Participants

The study was conducted in accordance with the Declaration of Helsinki and was approved by the local Research Ethics Committee. Written informed consent was obtained from all participants. Participants between 18 and 30 years of age completed the Beck Depression Inventory-II (BDI-II) (Beck et al. 1996, Sanz et al. 2003) and the Liebowitz Social Anxiety Scale (LSAS) (Liebowitz 1987, Bobes et al. 1999) on a website advertised through the university’s social networks. Potential participants were invited to a recruitment session where they were screened for depression, social anxiety, and other psychiatric symptoms using the Mini-International Neuropsychiatric Interview (MINI-Plus) (Sheehan et al. 1998, Ferrando et al. 2004). Two groups of participants were confirmed: a group with symptoms of major depression and/or social anxiety (MD-SA, *n* = 56) and a group of healthy controls (*n* = 47). Inclusion criteria for the MD-SA group were satisfying criteria for an episode of major depression and scoring ≥14 on the BDI-II and/or satisfying criteria for social anxiety and scoring ≥55 on the LSAS, and at least 3 weeks of not taking psychiatric medication. Controls had no current or past history of psychiatric disorders. Exclusion criteria for both groups were any neurological disorder and contraindication for MRI. The two groups were matched on the basis of gender, age, level of education, area of study, nicotine consumption, and dominant hand (Table 1). The MD-SA group was composed of 12 volunteers meeting criteria only for MD, 22 only for SA, and 22 for both disorders. During the recruitment session and between this session and the scanning day, participants completed several psychological questionnaires. See Table 1 and Supplementary material for details on these scales.

**Table 1.** Participant details.

Participant characteristics			
Demographic and psychological variables	Control group	MD-SA Group	*P*-value
*n*	47	56	
Age	23.76 ± 3.19	22.87 ± 3.04	.153
Sex (F/M)	18/29	14/42	.215
Nicotine consumption (no/yes)	43/3	49/7	.499
Dominant hand (right/left)	41/5	48/8	.633
Educational level (finished secondary studies, unfinished undergraduate studies, finished undergraduate studies, unfinished postgraduate studies, finished postgraduate studies)	1/40/4/1/1	1/49/5/0/1	.870
Area of studies (health, social sciences and arts, technology and science)	18/08/19	31/10/13	.134
BDI	1.53 ± 1.66	23.14 ± 10.07	<.001
LSAS total score	14.2 ± 10.27	75.75 ± 26.02	<.001
BAS	41.71 ± 5.78	38.78 ± 5.73	.012
BIS	18.1 ± 3.33	25 ± 2.68	<.001
FNE	7.45 ± 6.13	23.81 ± 5.91	<.001
TEPS anticipatory	41 ± 6.16	40.05 ± 7.29	.481
TEPS consummatory	38 ± 6.19	36.87 ± 6.7	.328
CBAS total score	42.67 ± 11.72	80.6 ± 18.61	<.001
ACIPS	89.91 ± 8.15	73.78 ± 13.66	.003
GASP negative behavior evaluation	3.98 ± 2.1	2.8 ± 1.96	.003
GASP guilt repair	4.17 ± 2.27	3.054 ± 1.9	.009
GASP negative autoevaluation	3.98 ± 1.73	2.78 ± 2.07	.001
GASP shame withdrawal	8.93 ± 11.89	5.02 ± 8.39	.065
Rosenberg	5.65 ± 3.99	18.01 ± 5.67	<.001
ZKPQ neuroticism-anxiety	1.78 ± 1.74	6.03 ± 3.6	<.001
ZKPQ impulsive sensation seeking	5.21 ± 2.71	3.51 ± 2.75	.011
ZKPQ activity	3.89 ± 2.86	2.35 ± 2.59	.015
ZKPQ sociability	5.46 ± 3.02	2.28 ± 2.09	<.001
ZKPQ aggression-hostility	2.89 ± 2.01	3.46 ± 2.84	.237

Values are given as mean ± standard deviation. *P*-values are based on the independent-samples *t*-test. BDI-II scores are taken from the experimental session. BIS/BAS: Behavioral Inhibition-Behavioral Activation Scale; TEPS: Temporal Experience of Pleasure Scale; GASP: Guilt and Shame Proneness Scale; ZKPQ: Zuckerman–Kuhlman Personality Questionnaire.

### Social comparison task

**Figure 1.**
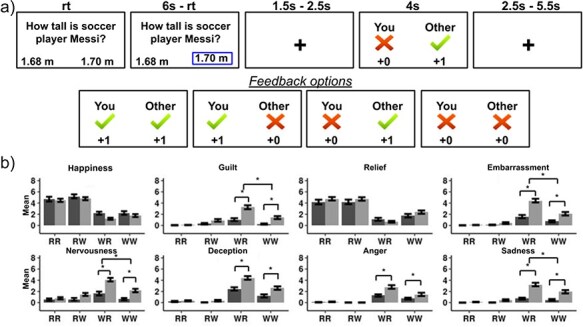
(a) The social comparison task. rt, reaction time; s, seconds. (b) Emotional responses to the social comparison task outcomes. RR: “Self_Right, Other_Right”; RW: “Self_Right, Other Wrong”; WR: “Self_Wrong, Other_Right”; WW: “Self_Wrong, Other Wrong’’. Error bars denote standard error of the mean. Asterisks denote significant between group differences.

During scanning participants performed a social comparison task similar to those previously used in the literature (Fig. 1a) (Boksem et al. 2011, Lindner et al. 2015). Before scanning, participants were instructed on how to perform the task. They were told that they would be playing a game with a coplayer connected through a computer network. In reality, the task was preprogrammed and the coplayer was not real. This procedure was implemented to standardize the feedback of the task across participants while preserving the social experience.

Participants were told that on each trial of the task, both they and the coplayer would be presented with a general knowledge question, accompanied by two possible answers. They would have to decide which was the correct answer. After responding, both the participant and the coplayer would receive feedback on each other’s performances. There were four possible feedback conditions: “Self_Right, Other_Right,” “Self_Right, Other_Wrong,” “Self_Wrong, Other_Right,” and “Self_Wrong, Other_Wrong.” Right/Wrong answers gave one/zero points. It was made clear to the participant that the performance of the coplayer had no influence on his/her outcome, and vice versa. Participants were told that both they and the coplayer would receive at the end of the game a nonmonetary reward according to the points they had accumulated during the task. Participants were shown a video recording with the supposed coplayer (an individual of the same sex as the participant) saluting them. They were told that after the scan they would meet the coplayer through a Zoom session and have the opportunity to talk about the game.

The task was programmed to provide the same feedback (independently of participants’ choices) to all participants. Each type of feedback was set up to appear on 25% of the trials. In case of missing responses, the feedback related to the participant’s performance was always Wrong, while the feedback for the coplayer could be either Right or Wrong. Missing responses were managed this way to motivate participants to always make a response (only 0.5% of responses were missing responses). In order to avoid suspicions about the veracity of the feedback (i.e. the participant knowing the correct answer to a question and receiving a wrong feedback), we used questions with a high level of difficulty. To achieve this, questions were designed with very close possible answers (e.g. “How tall is soccer player Messi?”; options: 1.68 m or 1.70 m). To prevent participants from feeling discouraged about the task and to give them some sense of control, a small set (eight in total) of easy questions was included. The feedback provided in these questions related to the actual performance of the participant with the coplayer always being right. On average, participants answered 92.85% of the easy questions correctly, while only 50.05% of the difficult questions were answered correctly. This led to a significant difference in the number of questions answered correctly depending on whether they were easy or difficult (*χ*^2^ = 42.3; *P* < .001). After participants were explained the task, they had a short practice on a computer and then completed a true/false questionnaire to ensure they understood the task.

In the scanner, participants played two sessions of the task. Each session contained 40 trials of difficult questions (10 per condition) plus 4 easy questions, lasting ∼11 min. The task was programmed on PsychoPy2 (version 1.84.2). At the end of the experiment, participants were debriefed regarding the cover story. Only one participant reported not believing the cover story and was excluded from analysis. All participants received the same reward (a decorated mug) in gratitude for their participation. During scanning, participants also completed another social task that will be reported elsewhere. This other social task was performed prior to the social comparison task in all participants.

### Emotional reports

After completing the task in the scanner, participants rated their emotional reactions toward the four feedback conditions of the task. They rated on a 0–8 Likert scale how much happiness, relief, embarrassment, nervousness, disappointment, sadness, anger, and guilt they felt when receiving each feedback.

For each emotion, the reports given by participants were fitted to a general linear model, using the glmer function (lme4 package) implemented in R (version 4.0.5), with self-outcome (participant right or wrong), other-outcome (coplayer right or wrong), and group as fixed effects, and subject as a random effect. Wald *F* tests were used to evaluate the significance of main effects and interactions. When *post hoc* paired *t*-tests were computed, we applied Tukey’s correction for multiple comparisons.

### fMRI image acquisition and data analyses

T2*-weighted gradient echo planar images were acquired using a 3T, GE Discovery 750 W MRI scanner with a 24-channel head coil. SPM12 (Statistical Parametric Mapping, http://www.fil.ion.ucl.ac.uk/spm) was used for analyses. Two hundred and seventy-five volumes were obtained with a repetition time of 2.5 s, an echo time of 30 ms, a flip angle of 90°, a field of view of 224 mm, and a 64 × 64 matrix. The first four volumes were discarded to allow for scanner transient effects. The first image from each session was aligned to the first scan of the first session. Then the images from each session were aligned to the first image of the session. The average realigned image was used to derive parameters for spatial normalization to the SPM12 Montreal Neurological Institute template with the parameters applied to each image in the time series. The realigned and spatially normalized images were then smoothed with an 8-mm full-width at half-maximum Gaussian kernel.

For the first-level analysis, an event-related design was used, which modeled blood-oxygen-level-dependent response at the feedback time. Four regressors of interest with a duration of 4 s convolved with the SPM12 canonical hemodynamic response function were defined, one for each task condition. In addition, a regressor representing the moment where the participant saw the general knowledge question (duration 6 s) and the six head motion realignment parameter estimates were included as covariates of no interest.

To detect brain regions involved in downward comparison, we analyzed the contrast [(Self_Right, Other_Wrong) > (Self_Right, Other_Right)]. For upward comparison, the [(Self_Wrong, Other_Right) > (Self_Wrong, Other_Wrong)] contrast was used. Contrast images of interest were taken to second-level analyses and within and between group activations explored using one-sample and two-sample *t*-tests.

For the transdiagnostic dimensional analysis of brain activity, first we applied a PCA to the scores of the psychological questionnaires used in the study to describe the psychological characteristics of the sample in a more compact low-dimensional manner. In order to study the relationship between brain activity from a dimensional perspective, contrast images of interest were taken to second-level analyses, and the scores of the first (that accounted for most of the variance, see later) component of the PCA on clinical ratings were added as a covariate in linear regression analyses.

Regions are reported as significant using a threshold of *P* < .05 corrected at the cluster level for multiple comparisons across the whole brain. This was achieved by a simultaneous requirement for a voxel threshold of *P* < .01 and a minimum cluster size of 166 continuous voxels. These parameters were obtained using a Monte Carlo method (Slotnick et al. 2003) that simulates whole-brain fMRI activation, assumes a type I error voxel activation based on the voxel threshold, smoothes the volume with a Gaussian kernel, and then counts the number of voxel clusters of a given size. After running a number of iterations (we implemented 10 000 iterations), the algorithm calculates a probability associated with each cluster extent, and the cluster extent threshold that yields the desired correction for multiple comparisons can be chosen.

## Results

Three data sets were excluded from all analyses, two due to hardware failure during fMRI data acquisition and one for not believing the cover story of the task. In addition, two data sets were excluded from the imaging analysis only due to technical problems in the fMRI images.

### Emotional results

See Fig. 1b for emotional findings. A significant effect of self-outcome was found with participants reporting higher levels of happiness (*F*(1297) = 53.99, *P* < .001) and relief (*F*(1294) = 67.30, *P* < .001), and lower levels of guilt (*F*(1297) = 13.26, *P* < .001), disappointment (*F*(1.297) = 67.80, *P* < .001), anger (*F*(1.297) = 28.62, *P* < .001), sadness (*F*(1297) = 12.13, *P* < .001), embarrassment (*F*(1297) = 34.20, *P* < .001), and nervousness (*F*(1297) = 18.71, *P* < .001) when they performed right compared to when they were wrong.

There was also a significant self-outcome × other-outcome interaction for the emotions of guilt (*F*(1, 297)= 7.24, *P* = .008), disappointment (*F*(1297) = 8.16, *P* = .046), embarrassment (*F*(1297) = 5.33, *P* = .002), and nervousness (*F*(1297) = 7.67, *P* < .006). *Posthoc* pairwise comparisons identified that when the participant was right, if the coplayer was right there were lower levels of guilt (*P* = .015) and similar levels of disappointment, embarrassment, and nervousness than if the coplayer was wrong. However, when the participant was wrong, a right answer of the coplayer elicited higher levels of guilt (*P* < .001), disappointment (*P* < .001), embarrassment (*P* < .001), and nervousness (*P* < .001) than a wrong answer of the coplayer. These results show that emotions were modulated by social comparison.

A significant self-outcome × group interaction was found for the emotions of guilt (*F*(1297) = 39.07, *P* < .001), disappointment (*F*(1.297) = 20.35, *P* < .001), anger (*F*(1297) = 15.64, *P* < .001), sadness (*F*(1297) = 51.24, *P* < .001), embarrassment (*F*(1297) = 48.08 *P* < .001), and nervousness (*F*(1297) = 36.32, *P* < .001). For these emotions, when the participant was right, there were no between group differences. However, when the participant was wrong, the MD-SA group reported higher levels of guilt (*P* < .001), disappointment (*P* < .001), anger (*P* < .001), sadness (*P* < .001), and embarrassment (*P* < .001) than the control group.

A significant self-outcome × other-outcome × group interaction was found for the emotions of guilt (*F*(1297) = 11.29, *P* < .001), sadness (*F*(1297) = 7.81, *P* = .006), embarrassment (*F*(1297)= 10.25, *P* = .002), and nervousness (*F*(1297) = 9.74, *P* < .002). For these emotions, when the participant was right, there were no between group differences independently of the coplayer response. However, when the participant was wrong, the MD-SA group reported higher levels of guilt, sadness, embarrassment, and nervousness than the control group, both when the coplayer was right (all *P* < .001) and when the coplayer was wrong (all *P* < .001), with between group differences being more accentuated for the upward comparison (“Self wrong, Other right”) than for the even wrong comparison (“Self wrong, Other wrong”) (all *P* ≤ .01). In summary, participants with depression and social anxiety symptoms did not differentiate from controls in emotional reports when performing correctly. However, when they were wrong, they reported higher negative feelings than controls, with the difference being enhanced for the upward comparison.

### Neuroimaging results

#### Activations related to downward comparisons

**Figure 2.**
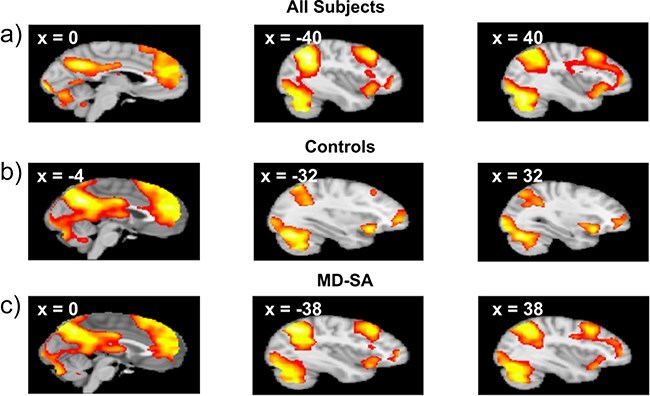
Neural responses related to downward comparison. Across all subjects (a) as well as in the control (b) and MD-SA (c) groups, the contrast for downward comparison elicited activations across the mPFC, dlPFC, posteromedial cortex, thalamus, caudate, and bilateral insula.

Across all participants, as well as within the control and MD-SA groups, downward comparison vs. the even right outcome ([(Self_Right, Other_Wrong) > (Self_Right, Other_Right)] elicited widespread activations across the mPFC, dorsolateral PFC (dlPFC), posteromedial cortex, thalamus, caudate, and bilateral anterior insula (Fig. 2, Supplementary Table S2). No significant between group (control vs. MD-SA) differences were observed.

#### Activations related to upward comparisons

**Figure 3.**
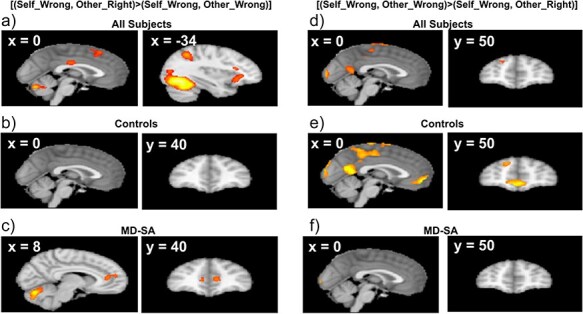
Neural responses related to upward comparison. For the contrast upward comparison greater than the even wrong outcome, (a) across all subjects activations were observed in the upper dmPFC and insula; (c) the MD-SA group showed activation in the dmPFC, (b) while controls did not show this activation. For the opposite contrast (the even wrong outcome greater than upward comparison), activations are shown across (d) all subjects; (e) controls; and (f) MD-SA group. Controls showed activation in the vmPFC and posteromedial cortex (e), while the MD-SA group did not show these activations (f).

The contrast for the upward comparison vs. the even wrong outcome ([(Self_Wrong, Other_Right) > (Self_Wrong, Other_Wrong)] showed activations in the upper dmPFC, dlPFC, and bilateral anterior insula across all participants (Fig. 3a, Supplementary Table S3). Interestingly, for this contrast there was an activation that was unique to the MD-SA group in the dmPFC extending into the ACC (Fig. 3e). For the opposite contrast, controls showed activations in the vmPFC and posteromedial cortex with these activations not being present in the MD-SA group (Fig. 3d and f).

**Figure 4.**
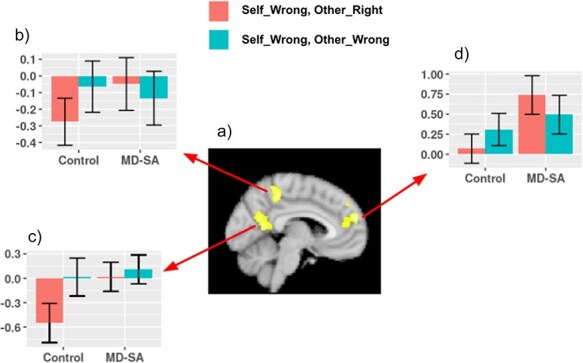
(a) Between group differences in neural responses related to upward comparison. In the bar graphs, the *Y*-axis represents the mean value of parameter estimates across voxels within a sphere diameter of 10 mm centered at peak coordinates (b) (−10, −42, 46), (c) (−4, −56, 14), and (d) (6, 56, 20). Error bars denote standard error of the mean.

Between group comparisons (Fig. 4, Supplementary Table S3) showed significant differences in the dmPFC and in the posteromedial cortex. In the dmPFC, this was driven by MD-SA participants having a stronger activation for the upward comparison outcome. In the posteromedial regions, the difference was driven by controls de-activating (decreasing activation below baseline) these regions for the upward comparison outcome, with the MD-SA group not showing this deactivation.

#### Dimensional analysis

The PCA analysis resulted in one component that accounted for 81% of the variance (see Supplementary material for the scree plot). This component loaded specially on the LSAS, the Cognitive-Behavioral Avoidance Scale (CBAS), the BDI, the Anticipatory and Consummatory Interpersonal Pleasure Scale (ACIPS), the Fear of Negative Evaluation (FNE), and the Rosenberg Self-Esteem Scale (RSES). Therefore, it relates to social anxiety, avoidance, and fear of negative evaluation, as well as to depression, social anhedonia, and low self-esteem (see the Supplementary material for the complete list of the questionnaires loadings on the first component of the PCA).The dimensional analysis using the main component of the PCA on the psychological questionnaires replicated the between group brain differences for upward comparison. Higher scores in the main component of the PCA correlated significantly with greater activity in the dmPFC and retrosplenial cortex for the upward comparison vs. the even wrong outcome contrast (Supplementary Fig. S2a). In addition, we tested for correlations between brain activity for this contrast and BDI-II and LSAS scores. To a lower level of significance (voxel threshold of *P* < .01 with no cluster threshold), the between group differences in the dmPFC and posteromedial cortex were again observed (Supplementary Fig. S2b–c).

## Discussion

This study investigated the neural basis of social comparison in participants with symptoms of MD-SA and healthy controls. Across subjects, participants reported higher/lower levels of positive/negative emotions when they performed right vs. when they did wrong. More importantly, participants’ emotions were modulated not only by their performance but also with the interaction between theirs and the coplayer’s performance. When participants were right, they reported similar levels of embarrassment, disappointment, and nervousness no matter the coplayer’s outcome. However, when participants were wrong, they reported higher levels of these negative emotions when the coplayer was right (i.e. for upward comparison) than for the even wrong outcome. For the emotion of guilt, the participant being right and coplayer wrong (i.e. downward comparison) elicited higher levels of this emotion than the even right outcome, while upward comparison elicited higher guilt than the even wrong outcome.

These findings indicate that the task was successful in eliciting social comparison processes. In particular, findings are in agreement with the notion that upward comparison can lead to negative emotions (Luo et al. 2018). The guilt findings indicate that not only upward but also downward comparison can be associated with this emotion, which is consistent with the proposal that, depending on the context, downward comparisons can elicit both positive and negative emotions (Buunk and Gibbons 2007).

Crucially, the emotional social comparison effects were modulated by group. When participants were right, there were not between group differences in emotions regardless of the coplayer outcome. However, when participants were wrong, the MD-SA group reported higher levels of negative emotions (guilt, sadness, embarrassment, and nervousness) than the control group, with this effect being enhanced for the upward comparison outcome. This is in agreement with proposals that upward comparison can be particularly challenging for people with depression and social anxiety eliciting stronger negative emotions (Bazner et al. 2006, McCarthy and Morina 2020, Uriarte-Gaspari et al. 2022). Overall, results are in agreement with theories that posit social comparison as a significant contributor to the development and maintenance of these disorders, triggering negative evaluations about the self (Swallow and Kuiper 1988, Antony et al. 2005).

Regarding the imaging findings, across participants downward comparison relative to the even right outcome elicited activations in the mPFC, striatum, posteromedial cortex, and salience network regions (dmPFC and bilateral insula). The upward comparison vs. the even wrong outcome also engaged the salience network. These results are consistent with previous findings (Kedia et al. 2014, Luo et al. 2018). Activations for downward comparisons in the striatum and mPFC have been related to reward and mentalizing processes (Fliessbach et al. 2007, Bault et al. 2011), whereas activations in the salience network for upward comparisons have been associated with the experience of conflict, negative affect, and salience detection (Swencionis and Fiske 2014, Lindner et al. 2015, Luo et al. 2018). Interestingly, in our study not only upward but also downward comparisons activated the salience network. This likely reflects that in the context of our task, both upward and downward comparisons represented salient situations when contrasted with the even outcomes.

Importantly, MD-SA participants differentiated from controls when contrasting brain activity for upward comparison relative to the even wrong outcome. Specifically, the MD-SA group showed stronger activations in the dmPFC and posteromedial cortex, regions of the DMN. In the dmPFC, the group difference was driven by a strong activation in MD-SA participants for upward comparison. In the posteromedial cortex, the group difference was driven by a deactivation in controls for upward comparison that was not present in MD-SA participants.

The dmPFC has been linked to self-referential thoughts (Northoff 2016, Davey and Harrison 2022), social cognition processes such as monitoring other’s mental states (Schurz et al. 2014), and rumination (Zhou et al. 2020). In this context, our finding of an enhanced activation in this region for upward comparison in MD-SA participants suggests increased self-referential processing, inferences about other’s mental states, and rumination in situations where they are outperformed by others.

The posteromedial cortex has also been linked to internally oriented processes such as self-referential processing (Whitfield-Gabrieli et al. 2011, Davey and Harrison 2022), social cognition (Menon 2023), memory (Cooper and Ritchey 2019), and rumination (Zhou et al. 2020). While controls deactivated this region for upward comparison, MD-SA participants did not show this effect. As part of the DMN, the posteromedial cortex usually deactivates during cognitively demanding tasks that imply attention to external stimuli (Menon 2023). Therefore, the deactivation observed in controls suggests that for these participants upward comparison is a salient event that deserves attention. In the case of MD-SA participants, while emotional reports indicate that upward comparison is a relevant event for them, the lack of deactivation in the posteromedial cortex suggests difficulties in disengaging from internally directed thinking, which may involve self-referential processing, inferences about other people thoughts, memories, and rumination.

Our findings of an increased DMN activity in participants with symptoms of depression and social anxiety are consistent with previous studies. In the case of depression, either more activation or less deactivation of DMN regions has been observed during the processing of sad words (Mitterschiffthaler et al. 2008), negative distractors (Kaiser et al. 2015), and negative pictures (Sheline et al. 2009), as well as in the context of self-focus (Lemogne et al. 2012) and emotional (Grimm et al. 2009, Zhang et al. 2017) tasks. In social anxiety, increased levels of DMN activity has been observed in response to self-referential criticism (Blair et al. 2008), unintentional transgressions (Blair et al. 2010), self-referential comments (Blair et al. 2011), self-focused attention (Boehme et al. 2015), eye contact (Schneier et al. 2011), emotional stimuli (Gentili et al. 2009), disorder-related visual scenes (Heitmann et al. 2016), and memory encoding (Yoon et al. 2016).

According to some studies, the DMN is composed of different subsystems (Andrews-Hanna et al. 2010, Christoff et al. 2016). A ‘core’ DMN subsystem is thought to be anchored on the vmPFC and the posteromedial cortex, while a ‘dorsal medial subsystem’ includes the dmPFC. Our findings suggest that depression and social anxiety interacted differently with these DMN subsystems during upward comparison, increasing activity in the dorsal medial subsystem and precluding deactivation in the core subsystem. This is in line with a revision of self-focus studies (Lemogne et al. 2012) in depression, which suggested that depression is associated with increased activity in the dmPFC and reduced deactivation in the vmPFC.

Interestingly, it has been proposed that internalizing disorders such as depression and social anxiety are associated with abnormal dynamics between neural networks, biasing resources to the internal vs. the external world (Fossati 2019, Yoon et al. 2019). It has been argued (Fossati 2019) that interpersonal stressors represent important factors for studying this altered allocation of resources in favor of the internal world. In the case of our study, the interpersonal stressor of interest is social comparison. Our findings suggest that people with depression and social anxiety respond to upward comparison by engaging in introspective, self-focus, and ruminative thinking that may exacerbate the negative emotional experience.

Limitations of the study should be noted. The sample was based on a university population, so caution should be taken in generalizing results to other populations. While we did find between group emotional and imaging differences for upward comparisons, we did not detect between-group differences for downward comparisons. In particular, it was expected that MD-SA participants would show decreased activations in reward regions for downward comparison, which was not observed. Alterations in the DMN could be expected as well. It would be worth it to explore this further, either with more severe or larger samples. Finally, the study sample (with a small number of participants that met criteria for only depression or only social anxiety) does not allow to investigate between-diagnosis differences.

In conclusion, this is one of the first studies investigating the neural underpinning of social comparison processes in depression and social anxiety. We found that the challenge of upward social comparison (not being as good as others) is experienced with higher levels of negative emotions in these disorders and that this is accompanied at the neural level with increased activation in DMN regions linked to self-focusing, inferences about other people’s thoughts, and rumination.

## Supplementary Material

nsaf012_Supp

## Data Availability

The data underlying this article cannot be shared publicly because it would require participants to sign an additional consent form, which is not possible to implement at the moment. Data will be shared on reasonable request to the corresponding author.

## References

[R1] Alvi T . Social Anxiety, Depression, and Emotional Congruence. 2020. https://scholar.smu.edu/hum_sci_psychology_etds/19/

[R2] Andrews-Hanna JR, Reidler JS, Sepulcre J et al. Functional-anatomic fractionation of the brain’s default network. *Neuron* 2010;65:550–62. doi: 10.1016/j.neuron.2010.02.005 20188659 PMC2848443

[R3] Antony MM, Rowa K, Liss A et al. Social comparison processes in social phobia. *Behav Ther* 2005;36:65–75. doi: 10.1016/S0005-7894(05)80055-3

[R4] Arditte Hall KA, Quinn ME, Vanderlind WM et al. Comparing cognitive styles in social anxiety and major depressive disorders: an examination of rumination, worry, and reappraisal. *Br J Clin Psychol* 2019;58:231–44. doi: 10.1111/bjc.12210 30484868 PMC6470033

[R5] Bault N, Joffily M, Rustichini A et al. Medial prefrontal cortex and striatum mediate the influence of social comparison on the decision process. *Proc Natl Acad Sci* 2011;108:16044–49. doi: 10.1073/pnas.1100892108 21896760 PMC3179055

[R6] Bazner E, Bromer P, Hammelstein P et al. Current and former depression and their relationship to the effects of social comparison processes. Results of an internet based study. *J Affect Disord* 2006;93:97–103. doi: 10.1016/j.jad.2006.02.017 16563520

[R7] Beck AT, Rush AJ, Shaw BF et al. *Cognitive Therapy of Depression*. New York: Guilford Press, 1979.

[R8] Beck AT, Steer RA, Ball R et al. Comparison of Beck Depression Inventories-IA and -II in psychiatric outpatients. *J Pers Assess* 1996;67:588–97. doi: 10.1207/s15327752jpa6703_13 8991972

[R9] Becker MPI, Simon D, Miltner WHR et al. Altered activation of the ventral striatum under performance-related observation in social anxiety disorder. *Psychol Med* 2017;47:2502–12. doi: 10.1017/S0033291717001076 28464974

[R10] Blair KS, Geraci M, Devido J et al. Neural response to self- and other referential praise and criticism in generalized social phobia. *Arch Gen Psychiatry* 2008;65:1176–84. doi: 10.1001/archpsyc.65.10.1176 18838634 PMC2785901

[R11] Blair KS, Geraci M, Hollon N et al. Social norm processing in adult social phobia: atypically increased ventromedial frontal cortex responsiveness to unintentional (embarrassing) transgressions. *Am J Psychiatry* 2010;167:1526–32. doi: 10.1176/APPI.AJP.2010.09121797 20889651 PMC3175630

[R12] Blair KS, Geraci M, Otero M et al. Atypical modulation of medial prefrontal cortex to self-referential comments in generalized social phobia. *Psychiatry Res Neuroim* 2011;193:38–45. doi: 10.1016/J.PSCYCHRESNS.2010.12.016 PMC3105197 21601433

[R13] Bobes J, Badía X, Luque A et al. [Validation of the Spanish version of the Liebowitz social anxiety scale, social anxiety and distress scale and Sheehan disability inventory for the evaluation of social phobia]. *Med Clin (Barc)* 1999;112:530–8. 10363239

[R14] Boehme S, Miltner WHR, Straube T. Neural correlates of self-focused attention in social anxiety. *Soc Cogn Affect Neurosci* 2015;10:856–62. doi: 10.1093/scan/nsu128 25326038 PMC4448029

[R15] Boehme S, Ritter V, Tefikow S et al. Brain activation during anticipatory anxiety in social anxiety disorder. *Soc Cogn Affect Neurosci* 2014;9:1413–18. doi: 10.1093/scan/nst129 23938870 PMC4158379

[R16] Boksem MAS, Kostermans E, De Cremer D. Failing where others have succeeded: Medial Frontal Negativity tracks failure in a social context. *Psychophysiology* 2011;48:973–79. doi: 10.1111/j.1469-8986.2010.01163.x 21175673

[R17] Borserio BJ, Sharpley CF, Bitsika V et al. Default mode network activity in depression subtypes. *Rev Neurosci* 2021;32:597–613. doi: 10.1515/revneuro-2020-0132 33583166

[R18] Borsini A, Wallis ASJ, Zunszain P et al. Characterizing anhedonia: a systematic review of neuroimaging across the subtypes of reward processing deficits in depression. *Cognit Affective Behav Neurosci* 2020;20:816–41. doi: 10.3758/s13415-020-00804-6 PMC7395022 32472419

[R19] Buunk AP, Brenninkmeyer V. Social comparison processes among depressed individuals: evidence for the evolutionary perspective on involuntary subordinate strategies? 2000.

[R20] Buunk AP, Gibbons FX. Social comparison: the end of a theory and the emergence of a field. *Organ Behav Hum Decis Process* 2007;102:3–21. doi: 10.1016/j.obhdp.2006.09.007

[R21] Carlton CN, Sullivan-Toole H, Ghane M et al. Reward circuitry and motivational deficits in social anxiety disorder: what can be learned from mouse models? *Front Neurosci* 2020;14:154. doi: 10.3389/fnins.2020.00154 PMC7054462 32174811

[R22] Christoff K, Irving Z, Fox K et al. Mind-wandering as spontaneous thought: a dynamic framework. *Nat Rev Neurosci* 2016;17:718–31. doi: 10.1038/nrn.2016.113 27654862

[R23] Clark DM, Wells A. A cognitive model of social phobia. In: *Social Phobia: Diagnosis, Assessment, and Treatment*. New York: The Guilford Press, 1995, 69–93.

[R24] Cooper RA, Ritchey M. Cortico-hippocampal network connections support the multidimensional quality of episodic memory. *eLife* 2019;8:e45591. doi: 10.7554/eLife.45591 PMC6450667 30900990

[R25] Cremers HR, Veer IM, Spinhoven P et al. Neural sensitivity to social reward and punishment anticipation in social anxiety disorder. *Front Behav Neurosci* 2015;8:439. doi: 10.3389/FNBEH.2014.00439 PMC4283602 25601830

[R26] Cuthbert BN . Research Domain Criteria: toward future psychiatric nosologies. *Dialogues Clin Neurosci* 2015;17:89–97. doi: 10.31887/DCNS.2015.17.1/bcuthbert 25987867 PMC4421905

[R27] Davey CG, Harrison BJ. The self on its axis: a framework for understanding depression. *Transl Psychiatry* 2022;12:Article1. doi: 10.1038/s41398-022-01790-8 PMC8766552 35042843

[R28] Disner SG, Beevers CG, Haigh EAP et al. Neural mechanisms of the cognitive model of depression. *Nat Rev Neurosci* 2011;12:467–77. doi: 10.1038/nrn3027 21731066

[R29] Dvash J, Gilam G, Ben-Ze’ev A et al. The envious brain: the neural basis of social comparison. *Human Brain Mapp* 2010;31:1741–50. doi: 10.1002/hbm.20972 PMC6871018 20205244

[R30] Fernández-Theoduloz G, Paz V, Nicolaisen-Sobesky E et al. Social avoidance in depression: a study using a social decision-making task. *J Abnormal Psychol* 2019;128:234–44. doi: 10.1037/abn0000415 30920233

[R31] Ferrando L, Bobes J, Gibert J. MINI Internation Neuropsychiatric Interviews. Versión en Español.. 2004.

[R32] Festinger L . A theory of social comparison processes. *Hum Relat* 1954;7:117–40. doi: 10.1177/001872675400700202

[R33] Fliessbach K, Weber B, Trautner P et al. Social comparison affects reward-related brain activity in the human ventral striatum. *Science* 2007;318:1305–08. doi: 10.1126/science.1145876 18033886

[R34] Fossati P . Circuit based anti-correlation, attention orienting, and major depression. *CNS Spectr* 2019;24:94–101. doi: 10.1017/S1092852918001402 30698129

[R35] Gentili C, Ricciardi E, Gobbini MI et al. Beyond amygdala: default mode network activity differs between patients with social phobia and healthy controls. *Brain Res Bull* 2009;79:409–13. doi: 10.1016/j.brainresbull.2009.02.002 19559343

[R36] Grimm S, Boesiger P, Beck J et al. Altered negative BOLD responses in the default-mode network during emotion processing in depressed subjects. *Neuropsychopharmacology* 2009;34:932–43. doi: 10.1038/npp.2008.81 18536699

[R37] Heitmann CY, Feldker K, Neumeister P et al. Abnormal brain activation and connectivity to standardized disorder-related visual scenes in social anxiety disorder. *Human Brain Mapp* 2016;37:1559–72. doi: 10.1002/hbm.23120 PMC6867294 26806013

[R38] Heitmann CY, Feldker K, Neumeister P et al. Brain activation to task-irrelevant disorder-related threat in social anxiety disorder: the impact of symptom severity. *NeuroImage Clin* 2017;14:323–33. doi: 10.1016/j.nicl.2017.01.020 28224080 PMC5310170

[R39] Kaiser RH, Andrews-Hanna JR, Spielberg JM et al. Distracted and down: neural mechanisms of affective interference in subclinical depression. *Soc Cogn Affect Neurosci* 2015;10:654–63. doi: 10.1093/scan/nsu100 25062838 PMC4420741

[R40] Kedia G, Mussweiler T, Linden DEJ. Brain mechanisms of social comparison and their influence on the reward system. *NeuroReport* 2014;25:1255–65. doi: 10.1097/WNR.0000000000000255 25191923 PMC4222713

[R41] Keren H, O’Callaghan G, Vidal-Ribas P et al. Reward processing in depression: a conceptual and meta-analytic review across fMRI and EEG studies. *Am J Psychiatry* 2018;175:1111–20. doi: 10.1176/appi.ajp.2018.17101124 29921146 PMC6345602

[R42] Koyuncu A, İnce E, Ertekin E et al. Comorbidity in social anxiety disorder: diagnostic and therapeutic challenges. *Drugs Context* 2019;8:1–13. doi: 10.7573/dic.212573 PMC6448478 30988687

[R43] Langer JK, Rodebaugh TL. Comorbidity of social anxiety disorder and depression. In: *The Oxford Handbook of Depression and Comorbidity* Oxford: Oxford University Press, 2014, 111–28.

[R44] Lemogne C, Delaveau P, Freton M et al. Medial prefrontal cortex and the self in major depression. *J Affect Disord* 2012;136:e1–11. doi: 10.1016/j.jad.2010.11.034 21185083

[R45] LeMoult J, Gotlib IH. Depression: a cognitive perspective. *Clinic Psychol Rev* 2019;69:51–66. doi: 10.1016/J.CPR.2018.06.008 PMC11884012 29961601

[R46] Liebowitz MR . Liebowitz Social Anxiety Scale. *Journal of Anxiety Disorders* 1987.

[R47] Lin H, Bruchmann M, Straube T. Altered putamen activation for social comparison-related feedback in social anxiety disorder: a pilot study. *Neuropsychobiology* 2023;82:359–72. doi: 10.1159/000531762 37717563

[R48] Lindner M, Rudorf S, Birg R et al. Neural patterns underlying social comparisons of personal performance. *Soc Cogn Affect Neurosci* 2015;10:569–76. doi: 10.1093/scan/nsu087 24948156 PMC4381240

[R49] Lorberbaum JP, Kose S, Johnson MR et al. Neural correlates of speech anticipatory anxiety in generalized social phobia. *Neuroreport* 2004;15:2701–05. 15597038

[R50] Luo Y, Eickhoff SB, Hétu S et al. Social comparison in the brain: a coordinate-based meta-analysis of functional brain imaging studies on the downward and upward comparisons. *Human Brain Mapp* 2018;39:440–58. doi: 10.1002/HBM.23854 PMC6866367 29064617

[R51] MacNamara A, Klumpp H, Kennedy AE et al. Transdiagnostic neural correlates of affective face processing in anxiety and depression. *Depression Anxiety* 2017;34:621–31. doi: 10.1002/da.22631 28453905 PMC5501757

[R52] McCarthy PA, Morina N. Exploring the association of social comparison with depression and anxiety: a systematic review and meta-analysis. *Clin Psychol Psychother* 2020;27:640–71. doi: 10.1002/cpp.2452 32222022

[R53] Menon V . 20 years of the default mode network: a review and synthesis. *Neuron* 2023;111:2469–87. doi: 10.1016/j.neuron.2023.04.023 37167968 PMC10524518

[R54] Mitterschiffthaler MT, Williams SCR, Walsh ND et al. Neural basis of the emotional Stroop interference effect in major depression. *Psychol Med* 2008;38:247–56. doi: 10.1017/S0033291707001523 17825123

[R55] Northoff G . Is the self a higher-order or fundamental function of the brain? The “basis model of self-specificity” and its encoding by the brain’s spontaneous activity. *Cogn Neurosci* 2016;7:203–22. doi: 10.1080/17588928.2015.1111868 26505808

[R56] Northoff G, Heinzel A, de Greck M et al. Self-referential processing in our brain—a meta-analysis of imaging studies on the self. *NeuroImage* 2006;31:440–57. doi: 10.1016/j.neuroimage.2005.12.002 16466680

[R57] Norton AR, Abbott MJ. Self-focused cognition in social anxiety: a review of the theoretical and empirical literature. *Behav Change* 2016;33:44–64. doi: 10.1017/bec.2016.2

[R58] Padmanabhan A, Lynch CJ, Schaer M et al. The default mode network in autism. *Biol Psych* 2017;2:476–86. doi: 10.1016/j.bpsc.2017.04.004 PMC5635856 29034353

[R59] Penney ES, Abbott MJ. Anticipatory and post-event rumination in social anxiety disorder: a review of the theoretical and empirical literature. *Behav Change* 2014;31:79–101. doi: 10.1017/bec.2014.3

[R60] Raichle ME . The brain’s default mode network. *Annu Rev Neurosci* 2015;38:433–47. doi: 10.1146/ANNUREV-NEURO-071013-014030 25938726

[R61] Richey JA, Ghane M, Valdespino A et al. Spatiotemporal dissociation of brain activity underlying threat and reward in social anxiety disorder. *Soc Cogn Affect Neurosci* 2017;12:81–94. doi: 10.1093/scan/nsw149 27798252 PMC5390704

[R62] Richey JA, Rittenberg A, Hughes L et al. Common and distinct neural features of social and non-social reward processing in autism and social anxiety disorder. *Soc Cogn Affect Neurosci* 2014;9:367–77. doi: 10.1093/scan/nss146 23223206 PMC3980795

[R63] Sanz J, Perdigón AL, Vázquez C. Adaptación española del Inventario para la Depresión de Beck-II (BDI-II): 2. Propiedades psicométricas en población general. *Clíni Y Salud* 2003;14:249–80.

[R64] Schneier FR, Pomplun M, Sy M et al. Neural response to eye contact and paroxetine treatment in generalized social anxiety disorder. *Psychiatry Res Neuroimaging* 2011;194:271–78. doi: 10.1016/j.pscychresns.2011.08.006 PMC3230304 22047726

[R65] Schurz M, Radua J, Aichhorn M et al. Fractionating theory of mind: a meta-analysis of functional brain imaging studies. *Neurosci Biobehav Rev* 2014;42:9–34. doi: 10.1016/j.neubiorev.2014.01.009 24486722

[R66] Sheehan DV, Lecrubier Y, Sheehan KH et al. The Mini-International Neuropsychiatric Interview (M.I.N.I.): the development and validation of a structured diagnostic psychiatric interview for DSM-IV and ICD-10. *J Clin Psychiatry* 1998;59:22–33. 9881538

[R67] Sheline YI, Barch DM, Price JL et al. The default mode network and self-referential processes in depression. *Proc Natl Acad Sci* 2009;106:1942–47. doi: 10.1073/pnas.0812686106 19171889 PMC2631078

[R68] Slotnick SD, Moo LR, Segal JB et al. Distinct prefrontal cortex activity associated with item memory and source memory for visual shapes. *Cognit Brain Res* 2003;17:75–82. doi: 10.1016/S0926-6410(03)00082-X 12763194

[R69] Swallow SR, Kuiper NA. Social comparison and negative self-evaluations: an application to depression. *Clinic Psychol Rev* 1988;8:55–76. doi: 10.1016/0272-7358(88)90049-9

[R70] Swencionis JK, Fiske ST. How social neuroscience can inform theories of social comparison. *Neuropsychologia* 2014;56:140–46. doi: 10.1016/j.neuropsychologia.2014.01.009 24486767 PMC4934127

[R71] Takahashi H, Kato M, Matsuura M et al. When your gain is my pain and your pain is my gain: neural correlates of envy and schadenfreude. *Science* 2009;323:937–39. doi: 10.1126/science.1165604 19213918

[R72] Uriarte-Gaspari L, Acuña A, Morales S et al. Who do I want in my team: social avoidance of high qualified partners in depression and social anxiety. *J Affect Disord Rep* 2022;10:100402. doi: 10.1016/j.jadr.2022.100402

[R73] Whitfield-Gabrieli S, Moran JM, Nieto-Castañón A et al. Associations and dissociations between default and self-reference networks in the human brain. *NeuroImage* 2011;55:225–32. doi: 10.1016/j.neuroimage.2010.11.048 21111832

[R74] Yoon H-J, Kim JS, Shin Y-B et al. Neural activity during self-referential working memory and the underlying role of the amygdala in social anxiety disorder. *Neurosci Lett* 2016;627:139–47. doi: 10.1016/j.neulet.2016.05.068 27260987

[R75] Yoon H-J, Seo EH, Kim JJ et al. Neural correlates of self-referential processing and their clinical implications in social anxiety disorder. *Clin Psychopharmacol Neurosci* 2019;17:12–24. doi: 10.9758/cpn.2019.17.1.12 30690936 PMC6361035

[R76] Zhang B, Li S, Zhuo C et al. Altered task-specific deactivation in the default mode network depends on valence in patients with major depressive disorder. *J Affect Disord* 2017;207:377–83. doi: 10.1016/j.jad.2016.08.042 27750155

[R77] Zhou HX, Chen X, Shen YQ et al. Rumination and the default mode network: meta-analysis of brain imaging studies and implications for depression. *NeuroImage* 2020;206:116287. doi: 10.1016/j.neuroimage.2019.116287 31655111

