# Supplementary material for: Increased default mode network activation in depression and social anxiety during upward social comparison
Source: Soc Cogn Affect Neurosci. 2025 Jan 30;20(1):nsaf012. doi: 10.1093/scan/nsaf012 (PMC11792650; doi:10.1093/scan/nsaf012)
Supplement: nsaf012_Supp [file nsaf012_supp.zip › scan-24-181-File006.docx]

**Supplementary Material**

*Clinical ratings:*

When participants registered for the study on the website of the study the completed the Beck Depression Inventory-II (BDI-II) [(Beck, Steer, Ball, & Ranieri, 1996; Sanz, Perdigón, & Vázquez, 2003)](https://www.zotero.org/google-docs/?7BgFAV) and the Liebowitz Social Anxiety Scale (LSAS) [(Bobes et al., 1999, p. 199; Liebowitz, 1987)](https://www.zotero.org/google-docs/?1wE1Jn). Later on during the recruitment session participants completed: the Behavioral Inhibition Behavioral Activation Scale (BIS/BAS)[(Carver & White, 1994; Segarra, Poy, López, & Moltó, 2014)](https://www.zotero.org/google-docs/?P4CKxg), a Brief Version of the Fear of Negative Evaluation (FNE) Scale [(Watson & Friend, 1969; Zubeidat, Salinas, & Sierra, 2007)](https://www.zotero.org/google-docs/?53cQ5P), The Temporal Experience of Pleasure Scale (TEPS) [(Gard, Gard, Kring, & John, 2006; Diane C Gooding, Fonseca-Pedrero, de Albéniz, Ortuño-Sierra, & Paino, 2016)](https://www.zotero.org/google-docs/?f0dUrw); The Cognitive-Behavioral Avoidance Scale (CBAS) [(Hernández-Guzmán et al., 2009; Ottenbreit & Dobson, 2004)](https://www.zotero.org/google-docs/?5cxmot), The Anticipatory and Consummatory Interpersonal Pleasure Scale (ACIPS) [(Diane C Gooding et al., 2016; Diane Carol Gooding & Pflum, 2014)](https://www.zotero.org/google-docs/?pQr0wj); the Guilt and Shame Proneness Scale (GASP) [(Alabèrnia-Segura, Feixas, & Gallardo-Pujol, 2018; Cohen, Wolf, Panter, & Insko, 2011)](https://www.zotero.org/google-docs/?tpyltR); and The Rosenberg Self-Esteem Scale (RSES) [(Rojas-Barahona, Zegers, & Förster, 2009; Rosenberg, 1965)](https://www.zotero.org/google-docs/?sbt1HO). Between the recruitment session and the scanning day, participants also completed the Zuckerman-Kuhlman Personality Questionnaire Form III [(Martín Poó, Daniel Ledesma, & López, 2013; Zuckerman, Kuhlman, Joireman, Teta, & Kraft, 1993)](https://www.zotero.org/google-docs/?E6JpbG). On the day of the scanning session, participants completed the BDI again. This measure of the BDI was the one included for the PCA analysis.

The BDI-II is one of the most widely used self-reported inventory for assessing depressive symptomatology [(Beck et al., 1996)](https://www.zotero.org/google-docs/?Wu9ET0). The LSAS is a self-rated scale that measures social phobia symptoms in two domains: fear/anxiety and avoidance [(Liebowitz, 1987)](https://www.zotero.org/google-docs/?Io8K7K). The BIS/BAS scale assesses individual differences in dispositions that reflect the sensitivity of a behavioral inhibition system that bears on aversive motivation, and of a behavioral activation system that bears on appetitive motivation [(Carver & White, 1994)](https://www.zotero.org/google-docs/?GZIHJi). The FNE assesses apprehension about others evaluations, distress about negative evaluations, avoidance of evaluative situations, and expectations about others evaluating oneself negatively [(Watson & Friend, 1969)](https://www.zotero.org/google-docs/?hUHNas). The TEPS measures individual dispositions in both anticipatory and consummatory experiences of pleasure [(Gard et al., 2006)](https://www.zotero.org/google-docs/?3PTUCC). The CBAS measures avoidance along cognitive/behavioral and social/nonsocial dimensions [(Ottenbreit & Dobson, 2004)](https://www.zotero.org/google-docs/?dt20nh). The ACIPS measures hedonic capacity for social and interpersonal pleasure [(Diane Carol Gooding & Pflum, 2014)](https://www.zotero.org/google-docs/?ecTQi2). The GASP assesses the propensity to experience guilt and shame across a range of personal transgressions [(Cohen et al., 2011)](https://www.zotero.org/google-docs/?xTIuFQ). The RSES assesses self-esteem [(Rosenberg, 1965)](https://www.zotero.org/google-docs/?9r3s7Z). The Zuckerman-Kuhlman Personality Questionnaire Form III measures personality traits along five dimensions: impulsive sensation seeking, neuroticism-anxiety, aggression-hostility, sociability and activity [(Zuckerman et al., 1993)](https://www.zotero.org/google-docs/?FGwwUX).

**Supplementary Figure 1.** Scree plot showing the eigenvalues of the PCA components.

**
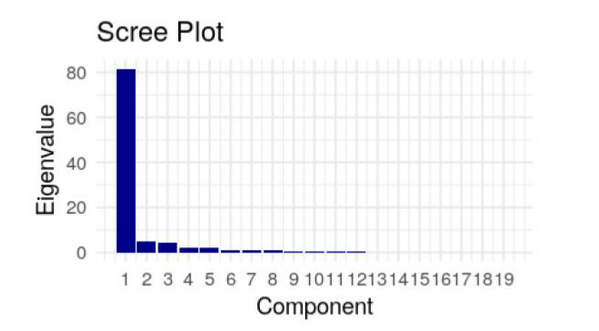
**

**Supplementary Table 1 -** Questionnaires loadings on the first component of the PCA

| **Questionnaire** | **Loading** |
| --- | --- |
| Beck Depression Inventory | 0.232 |
| Liebowitz Social Anxiety Scale | 0.771 |
| Behavioral Activation Scale | -0.040 |
| Behavioral Inhibition Scale | 0.070 |
| Fear of Negative Evaluation | 0.182 |
| Temporal Experience of Pleasure Scale: Subscale Anticipation | -0.027 |
| Temporal Experience of Pleasure Scale: Subscale Consummation | -0.021 |
| Cognitive Behavioral Avoidance Scale | 0.488 |
| Anticipatory and Consummatory Interpersonal Pleasure Scale | -0.219 |
| Guilt and Shame Proneness Scale: subscale Guilt‐Negative‐Behavior‐Evaluation | -0.015 |
| Guilt and Shame Proneness Scale: subscale Guilt‐Repair | -0.013 |
| Guilt and Shame Proneness Scale: subscale Shame‐Negative‐Self‐Evaluation | -0.014 |
| Guilt and Shame Proneness Scale: subscale Shame‐Withdraw | -0.006 |
| Rosenberg Self Esteem Questionnaire | -0.141 |
| ZKPQ Personality: subscale Neuroticism Anxiety | 0.046 |
| ZKPQ Personality: subscale Sociability | -0.037 |
| ZKPQ Personality: subscale Aggression-Hostility | 0.002 |
| ZKPQ Personality: subscale Impulsive Sensation Seeking | -0.015 |
| ZKPQ Personality: subscale Activity | -0.016 |

**Supplementary Table 2 Within group brain activations related to downward comparison.**

L/R, Left/Right; Coordinates (x,y,z) reported in MNI space. -a Indicates that the peak belongs to the same cluster as the peak above.

| **Region Name** | **Cluster size** | **X** | **Y** | **Z** | **T** |
| --- | --- | --- | --- | --- | --- |
| **Contrast: [(Self_Right, Other_Wrong)>(Self_Right, Other_Right)]** |  |  |  |  |  |
| **All Subjects** |  |  |  |  |  |
| amPFC | 64767 | -2 | 46 | 24 | 8.48 |
| dmPFC | -a | -8 | 24 | 60 | 6.67 |
| L dlPFC | -a | -42 | 24 | 44 | 8.81 |
| R dlPFC | -a | 52 | 24 | 40 | 6.58 |
| Posteromedial Cortex | -a | -2 | -58 | 36 | 9.54 |
| L Superior Parietal Lobe | -a | -42 | -54 | 42 | 9.64 |
| R Superior Parietal Lobe | -a | 44 | -56 | 54 | 8.03 |
| L Caudate | -a | -12 | 12 | 14 | 6.56 |
| R Caudate | -a | 12 | 12 | 10 | 6.89 |
| Thalamus | -a | -8 | -10 | 14 | 5.24 |
| L Anterior Insula | -a | -32 | 20 | -16 | 7.29 |
| R Anterior Insula | -a | 34 | 22 | -14 | 5.59 |
| L Cerebellum | -a | -32 | -74 | -24 | 9.40 |
| R Cerebellum | -a | 40 | -66 | -24 | 8.82 |
| **Control Group** |  |  |  |  |  |
| amPFC | 14215 | 0 | 52 | 26 | 5.92 |
| dmPFC | -a | -10 | 40 | 46 | 4.66 |
| L dlPFC | -a | -42 | 24 | 44 | 5.37 |
| L Caudate | -a | -10 | 14 | 16 | 4.80 |
| L Anterior Insula | -a | -30 | 22 | -14 | 5.22 |
| R Anterior Insula | -a | 30 | 20 | -12 | 5.45 |
| R dlPFC | 571 | 50 | 18 | 42 | 3.31 |
| L Cerebellum | 22299 | -24 | -80 | -24 | 7.70 |
| R Cerebellum | -a | 34 | -72 | -24 | 6.74 |
| Posteromedial Cortex | -a | -2 | -56 | 34 | 6.84 |
| L Superior Parietal Lobe | -a | -42 | -54 | 34 | 5.75 |
| R Superior Parietal Lobe | -a | 48 | -62 | 42 | 4.65 |
| R Caudate | 636 | 12 | 12 | 10 | 4.00 |
| Thalamus | -a | 8 | -10 | 16 | 3.11 |
| **MD-SA group** |  |  |  |  |  |
| amPFC | 52418 | 0 | 58 | 20 | 6.46 |
| dmPFC | -a | -4 | 24 | 60 | 6.13 |
| L dlPFC | -a | -42 | 24 | 42 | 7.09 |
| R dlPFC | -a | 46 | 24 | 44 | 6.66 |
| Posteromedial Cortex | -a | -2 | -60 | 42 | 7.68 |
| L Superior Parietal Lobe | -a | -50 | -56 | 40 | 8.86 |
| R Superior Parietal Lobe | -a | 44 | -54 | 48 | 7.00 |
| L Caudate | -a | -10 | 14 | 10 | 4.96 |
| R Caudate | -a | 12 | 14 | 12 | 6.04 |
| Thalamus | -a | 4 | -12 | 10 | 4.73 |
| L Anterior Insula | -a | -34 | 20 | -18 | 5.81 |
| L Cerebellum | -a | -34 | -74 | -24 | 6.58 |
| R Cerebellum | -a | 40 | -76 | -24 | 5.77 |
| R Anterior Insula | 1171 | 42 | 22 | -20 | 4.73 |
| **Contrast: [(Self_Right, Other_Right)>(Self_Right, Other_Wrong)]** |  |  |  |  |  |
| **All Subjects** |  |  |  |  |  |
| L Inferior Parietal Lobe | 748 | -64 | -26 | 34 | 4.60 |
| R Inferior Parietal Lobe | 292 | 66 | -28 | 38 | 4.18 |
| L Precuneus | 587 | -12 | -48 | 58 | 3.95 |
| R Precuneus | 1028 | 14 | -42 | 60 | 4.32 |
| L Superior Frontal Lobe | 307 | -18 | -4 | 70 | 3.72 |
| R Superior Frontal Lobe | 327 | 22 | -2 | 68 | 3.75 |
| **Control group** |  |  |  |  |  |
| L Precuneus | 179 | -12 | -56 | 56 | 3.19 |
| R Precuneus | 368 | 14 | -52 | 60 | 4.07 |
| R Posterior Cingulate Cortex | -a | 12 | -28 | 44 | 3.68 |
| L Posterior Cingulate Cortex | 217 | -10 | -30 | 46 | 3.95 |
| **MD-SA group** |  |  |  |  |  |
| L Inferior Parietal Lobe | 789 | -66 | -26 | 32 | 4.56 |
| R Inferior Parietal Lobe | 385 | 64 | -26 | 40 | 3.60 |
| **Between group differences**  **Contrast: [(Self_Right, Other_Wrong)>(Self_Right, Other_Right)]** |  | | | | |
| **MD-SA > Controls** | No significant differences were observed | | | | |
| **Controls > MD-SA** | No significant differences were observed | | | | |

**Supplementary Table 3 Within and between group brain activations related to upward comparison.**

L/R, Left/Right; Coordinates (x,y,z) reported in MNI space. -a Indicates that the peak belongs to the same cluster as the peak above.

| **Region Name** | **Cluster size** | **X** | **Y** | **Z** | **T** |
| --- | --- | --- | --- | --- | --- |
| **Contrast: [(Self_Wrong, Other_Right)>(Self_Wrong, Other_Wrong)]** |  |  |  |  |  |
| **All Subjects** |  |  |  |  |  |
| dmPFC | 207 | 0 | 26 | 56 | 3 |
| L Anterior Insula | 344 | -30 | 32 | 0 | 3.67 |
| R Anterior Insula | 394 | 38 | 24 | -10 | 4.62 |
| L dlPFC | 404 | -42 | 22 | 20 | 3.31 |
| Middle Cingulate Cortex | 176 | 4 | -20 | 32 | 3.16 |
| L Superior Parietal Cortex | 1199 | -26 | -54 | 46 | 5.46 |
| R Superior Parietal Cortex | 530 | 28 | -58 | 38 | 6.56 |
| Left Cerebellum | 3682 | -40 | -62 | -12 | 7.61 |
| Right Cerebellum | 3330 | 36 | -70 | -10 | 5.88 |
| **Controls** |  |  |  |  |  |
| L Superior Parietal Cortex | 881 | -28 | -60 | 48 | 4.21 |
| L Cerebellum | 1019 | -38 | -62 | -10 | 4.35 |
| R Cerebellum | 422 | 34 | -70 | -8 | 3.60 |
| **MD-SA** |  |  |  |  |  |
| amPFC/ACC | 311 | -12 | 30 | 16 | 3.23 |
| R Anterior Insula | 205 | 38 | 24 | -10 | 3.77 |
| L dlPFC | 308 | -56 | 28 | 18 | 3.33 |
| Middle Cingulate Cortex | 276 | 2 | -20 | 36 | 3.36 |
| L Superior Parietal Cortex | 580 | -26 | -52 | 48 | 4.41 |
| R Superior Parietal Cortex | 318 | 26 | -58 | 40 | 4.06 |
| L Cerebellum | 3784 | -38 | -62 | -16 | 6.72 |
| R Cerebellum | 3580 | 32 | -60 | -20 | 5.12 |
| **Contrast: [(Self_Wrong, Other_Wrong)>(Self_Wrong, Other_Right)]** |  |  |  |  |  |
| **All Subjects** |  |  |  |  |  |
| Posterior Cingulate Cortex / Retrosplenial Cortex | 1352 | -12 | -56 | 16 | 4.44 |
| Precuneus | 915 | 10 | -50 | 66 | 3.29 |
| L Occipital Cortex | 1984 | -12 | -96 | 6 | 6.86 |
| R Occipital Cortex | -a | 18 | -96 | 6 | 6.58 |
| L Angular Gyrus | 591 | -48 | -74 | 30 | 5.15 |
| R Angular Gyrus | 458 | 48 | -72 | 30 | 5.70 |
| L Middle Temporal Cortex | 1001 | -60 | 2 | -26 | 3.55 |
| R Middle Temporal Cortex | 1264 | 58 | -6 | -14 | 4.91 |
| L dlPFC | 1140 | -22 | 32 | 42 | 4.04 |
| R dlPFC | 1026 | 26 | 22 | 42 | 4.81 |
| Controls |  |  |  |  |  |
| vmPFC | 467 | 2 | 52 | -10 | 4.07 |
| Posterior Cingulate Cortex / Retrosplenial Cortex | 2051 | 14 | -52 | 12 | 5.41 |
| Precuneus | 2551 | 8 | -44 | 52 | 3.82 |
| L dlPFC | 1062 | -24 | 36 | 44 | 4.90 |
| R dlPFC | 470 | 28 | 24 | 42 | 4.45 |
| L Angular Gyrus | 600 | -46 | -72 | 36 | 4.04 |
| R Angular Gyrus | 642 | 50 | -72 | 30 | 4.88 |
| L Superior Temporal Cortex | 1890 | -56 | -12 | 2 | 4.59 |
| R Superior Temporal Cortex | 1872 | 64 | -18 | 16 | 4.26 |
| **Controls** |  |  |  |  |  |
| **MD-SA** |  |  |  |  |  |
| L Occipital Cortex | 651 | -14 | -96 | 2 | 5.53 |
| R Occipital Cortex | 375 | 18 | -96 | 4 | 5.24 |
| L dlPFC | 224 | -20 | 16 | 54 | 3.15 |
| R dlPFC | 514 | 24 | 18 | 44 | 3.95 |
| **Between group differences**  **Contrast: [(Self_Wrong, Other_Right)>(Self_Wrong, Other_Wrong)]** |  |  |  |  |  |
| **MD-SA > Controls** |  |  |  |  |  |
| amPFC | 515 | -10 | 58 | 20 | 3.37 |
| Precuneus | 450 | -10 | -42 | 46 | 3.45 |
| Posterior Cingulate Cortex / Retrosplenial Cortex | 477 | -4 | -56 | 14 | 3.09 |
| **Controls > MD-SA** | No significant activations were observed | | | | |
|  |  |  |  |  |  |

**
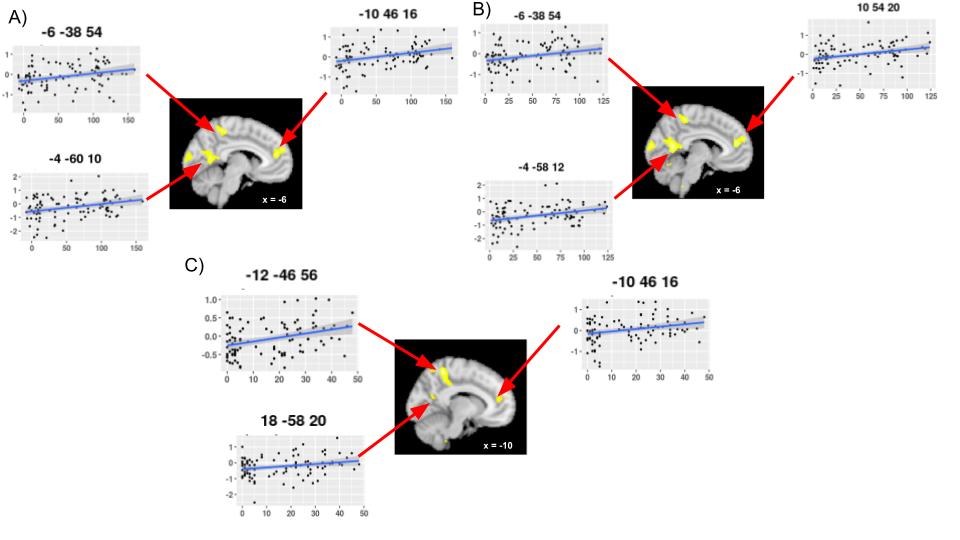
**

**Supplementary Figure 2**. A) Correlation between brain activity for the upward comparison contrast [(Self_Wrong, Other_Right)>(Self_Wrong, Other_Wrong)] and participant’s scores on the first component of the PCA. To a lower level of significance (voxel threshold of p<0.01 with no cluster threshold) correlations between brain activity for this contrast and LSAS B) and BDI-II C) scores also showed clusters in the dmPFC and posteromedial cortex. Scatter plots show mean value of parameter estimates across voxels within a sphere of diameter 10mm centered at peak coordinates of the corresponding regions.

**References**

[Alabèrnia-Segura, M., Feixas, G., & Gallardo-Pujol, D. (2018). *Guilt and Shame Proneness Scale (GASP) Adaptation and Psychometric Properties in Spanish Population*. Recuperado de https://europepmc.org/article/ppr/ppr333548](https://www.zotero.org/google-docs/?sGSeO5)

[Beck, A. T., Steer, R. A., Ball, R., & Ranieri, W. (1996). Comparison of Beck Depression Inventories -IA and -II in psychiatric outpatients. *Journal of personality assessment*, *67*(3), 588-597. https://doi.org/10.1207/s15327752jpa6703_13](https://www.zotero.org/google-docs/?sGSeO5)

[Bobes, J., Badia, X., Luque, A., Garcia, M., González, M. P., & Dal-Ré, R. (1999). Validación en Español de los Cuestionarios de Evaluación de la Fobia Social. Validación de las versiones en español de los cuestionarios Liebowitz Social Anxiety Scale, Social Anxiety and Distress Scale, y Sheehan Disability Inventory para la evaluación d. *Med Clin (Barc)*, *112*, 530-538.](https://www.zotero.org/google-docs/?sGSeO5)

[Carver, C. S., & White, T. L. (1994). Behavioral Inhibition, Behavioral Activation, and Affective Responses to Impending Reward and Punishment: The BIS/BAS Scales. *Journal of Personality and Social Psychology*, *67*(2), 319-333. https://doi.org/10.1037/0022-3514.67.2.319](https://www.zotero.org/google-docs/?sGSeO5)

[Cohen, T. R., Wolf, S. T., Panter, A. T., & Insko, C. A. (2011). Introducing the GASP scale: A new measure of guilt and shame proneness. *Journal of personality and social psychology*, *100*(5), 947.](https://www.zotero.org/google-docs/?sGSeO5)

[Gard, D. E., Gard, M. G., Kring, A. M., & John, O. P. (2006). Anticipatory and consummatory components of the experience of pleasure: A scale development study. *Journal of Research in Personality*, *40*(6), 1086-1102. https://doi.org/10.1016/J.JRP.2005.11.001](https://www.zotero.org/google-docs/?sGSeO5)

[Gooding, Diane C, Fonseca-Pedrero, E., de Albéniz, A. P., Ortuño-Sierra, J., & Paino, M. (2016). Adaptación española de la versión para adultos de la Escala de Placer Interpersonal Anticipatorio y Consumatorio. *Revista de Psiquiatr{\’\i}a y Salud Mental*, *9*(2), 70-77.](https://www.zotero.org/google-docs/?sGSeO5)

[Gooding, Diane Carol, & Pflum, M. J. (2014). The assessment of interpersonal pleasure: Introduction of the Anticipatory and Consummatory Interpersonal Pleasure Scale (ACIPS) and preliminary findings. *Psychiatry research*, *215*(1), 237-243.](https://www.zotero.org/google-docs/?sGSeO5)

[Hernández-Guzmán, L., Dobson, K. S., Caso-Niebla, J., González-Montesinos, M., Epp, A., Arratíbel-Siles, M. L., & Wierzbicka-Szymczak, E. (2009). The Spanish version of the Cognitive-Behavioral Avoidance Scale (CBAS). *Revista Latinoamericana de Psicologia*, *41*(1). Recuperado de http://www.scielo.org.co/scielo.php?pid=S0120-05342009000100008&script=sci_abstract&tlng=pt](https://www.zotero.org/google-docs/?sGSeO5)

[Liebowitz, M. R. (1987). *Social Phobia* (D. F. Klein, Ed.). En (pp. 141-173). S. Karger AG. https://doi.org/10.1159/000414022](https://www.zotero.org/google-docs/?sGSeO5)

[Martín Poó, F., Daniel Ledesma, R., & López, S. (2013). Versión transcultural del Cuestionario de Personalidad de Zuckerman-Kuhlman (ZKPQ-50-CC) en población argentina. *Escritos de Psicología (Internet)*, *6*(1), 1-5.](https://www.zotero.org/google-docs/?sGSeO5)

[Ottenbreit, N. D., & Dobson, K. S. (2004). Avoidance and depression: The construction of the Cognitive-Behavioral Avoidance Scale. *Behaviour Research and Therapy*, *42*(3), 293-313. https://doi.org/10.1016/S0005-7967(03)00140-2](https://www.zotero.org/google-docs/?sGSeO5)

[Rojas-Barahona, C. A., Zegers, B., & Förster, C. E. (2009). La escala de autoestima de Rosenberg: Validación para Chile en una muestra de jóvenes adultos, adultos y adultos mayores. *Revista médica de Chile*, *137*(6), 791-800. https://doi.org/10.4067/S0034-98872009000600009](https://www.zotero.org/google-docs/?sGSeO5)

[Rosenberg, M. (1965). *Society and the adolescent self-image*. Princeton NJ: Princeton University Press.](https://www.zotero.org/google-docs/?sGSeO5)

[Sanz, J., Perdigón, A. L., & Vázquez, C. (2003). Adaptación española del Inventario para la Depresión de Beck-II (BDI-II): 2. Propiedades psicométricas en población general. *Clínica y Salud*, *14*(3), 249-280.](https://www.zotero.org/google-docs/?sGSeO5)

[Segarra, P., Poy, R., López, R., & Moltó, J. (2014). Characterizing Carver and White’s BIS/BAS subscales using the Five Factor Model of personality. *Personality and Individual Differences*, *61-62*, 18-23. https://doi.org/10.1016/j.paid.2013.12.027](https://www.zotero.org/google-docs/?sGSeO5)

[Watson, D., & Friend, R. (1969). Measurement of Social-Evaluative Anxiety. *Journal of Consulting and Clinical Psychology*, *33*(4), 448-. https://doi.org/10.1037/h0027806](https://www.zotero.org/google-docs/?sGSeO5)

[Zubeidat, I., Salinas, J. M., & Sierra, J. C. (2007). Escala de Miedo a la Evaluación Negativa y Escala de Evitación y Malestar Social: Fiabilidad y validez en una muestra de adolescentes españoles. *Cl{\’\i}nica y Salud*, *18*(1), 57-81.](https://www.zotero.org/google-docs/?sGSeO5)

[Zuckerman, M., Kuhlman, D. M., Joireman, J., Teta, P., & Kraft, M. (1993). A comparison of three structural models for personality: The big three, the big five, and the alternative five. *Journal of personality and social psychology*, *65*(4), 757.](https://www.zotero.org/google-docs/?sGSeO5)
